# Fibular Bone Graft for Nasal Septal Reconstruction: A Case Report

**Published:** 2017-05

**Authors:** Yakup Cil

**Affiliations:** Diyarbakır Military Hospital, Department of Plastic Surgery 21000 Diyarbakır, Turkey

**Keywords:** Fibula, Bone graft, Nose deformity

## Abstract

Although various techniques have been described for treatment of severe nose deformities, these problems have high revision rates. Conventional nasal septal surgery may not be adequate for all cases. A 21-year-old male patient with nose deformity underwent a nasal surgery. Patient had both functional and aesthetic nasal problems. Rigid fibular bone graft was used for corrective nasal surgery. Duration of the operation was three hours. Patient recovered without problems. Aesthetic and functional results of the operation were acceptable. Fibular bone graft may offer a long lasting support in treatment of severe nose deformity.

## INTRODUCTION

Although various techniques have been described for nose deformity corrections,^1^^,^^2^ these problems still have high recurrence and revision rates. Conventional nasal septal surgery may not be adequate for preventing recurrences. In the management of this case; fibular bone graft have been used for corrective nasal surgery.^1^^,^^2^

## CASE REPORT

A 21-year-old male patient with nose deformity underwent a nasal surgery. Nasal deformity was secondary to congenital cleft lip-nose. He had been operated for cleft lip-nose in childhood. Patient had functional and cosmetic nasal problems when he admitted to our clinic. Physical examination showed that the nasal septum was severely distorted. We decided to use fibular bone graft for nasal septal reconstruction. After local anesthetic infiltration, nasal structures were exposed through an open rhinoplasty approach. Following hump resection, septum was dissected subperichondrially. 

**Fig. 1:**
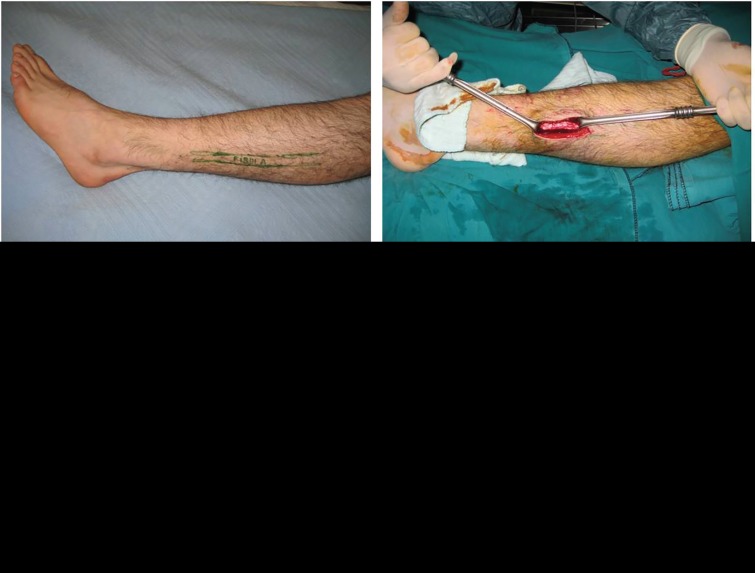
Bone graft was taken from the lateral side of fibular bone. Radiologic appearance of fibular bone graft donor site is also seen

**Fig. 2:**
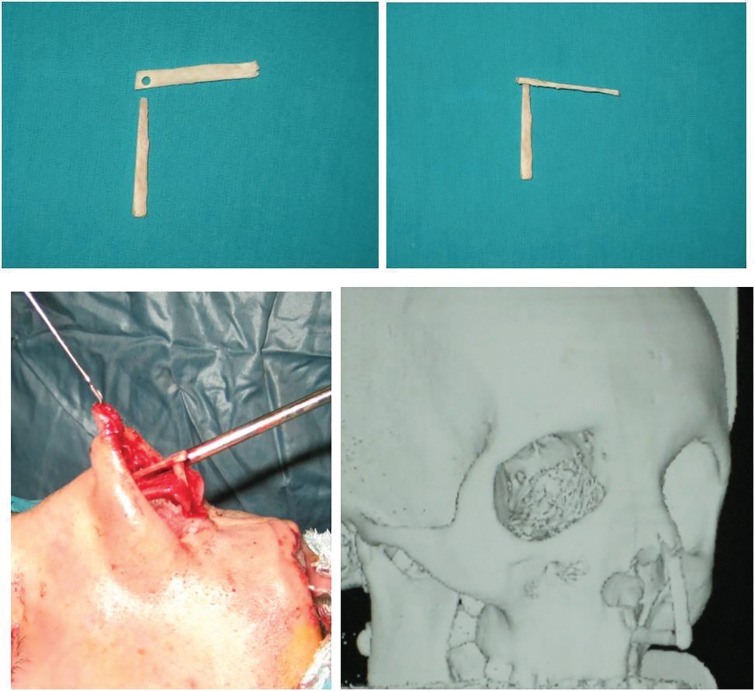
Two pieces of L-strut bone graft (above, left). A interlocked design (key in the keyhole pattern; above, right) L-strut bone graft was placed the nose (below, left). Three dimensional CT imaging of L-strut bone graft (below, right) is seen

Nasal septal cartilage was thick and it was distorted. Septal cartilage sculpturing was not possible and distorted cartilage septum was removed. Fibular bone graft was taken from lateral side of the fibula (Figure 1). Lateral leg muscles were protected during bone graft harvesting procedure. Bone graft was thinned by abrasion and shaped as L-strut as “key in the keyhole pattern” (Figure 2). The dorsal strut was placed as tongue in groove technique on the nasal bone. L-strut frame was sutured to upper lateral cartilage remnants and the domes of two lower lateral cartilages to hide palpable edges and secure it at its place. Lateral and median osteotomies were performed. Duration of the operation was three hours. After closure, nasal packing and plaster cast were applied. 

**Fig. 3:**
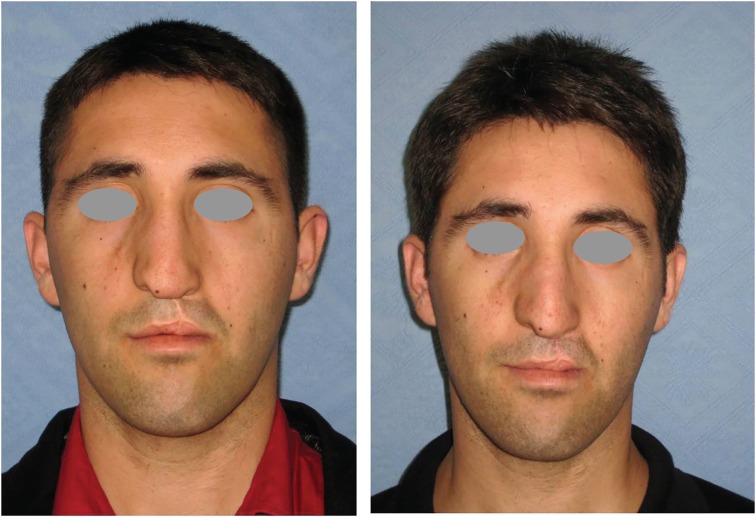
Preoperative anterior view of the patient with nose deformity (left). Postoperative twenty-four months later view (right

**Fig. 4:**
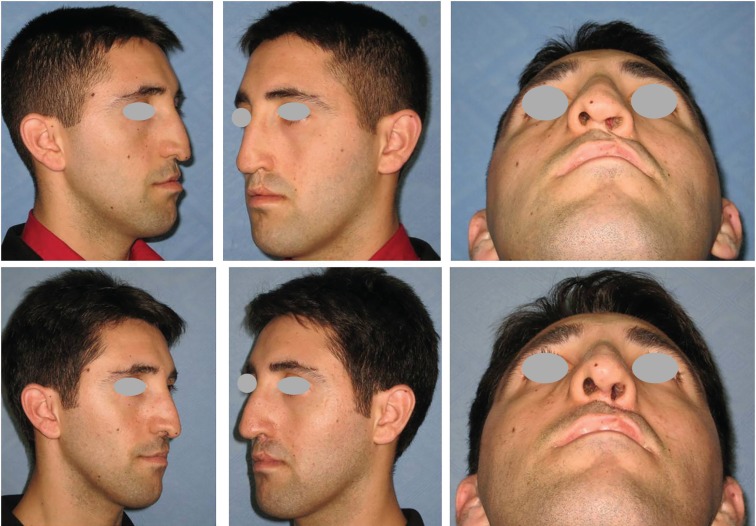
Preoperative oblique (above; left and center), inferior view (above, right) of the nose deformity. Postoperative twenty-four months oblique (below; left and center), inferior view (below, right

A plaster cast was also applied on the leg and removed 1 week later. The packing and the nasal splint were removed at 3th and 7th days, respectively. Patient has recovered without problems. Aesthetic and functional results of the operation were acceptable in aspect of patient’s satisfaction (Figures 3 and 4). The follow-up period was 24 months. The graft did not shift, and did not develop unsightly irregularities over time. No donor site complication has been observed.

## DISCUSSION

Nasal septum support is crucial for long lasting results for management of complicated nose reconstruction.^2^ The most popular method is reinforcement of corrected septum with spreader grafts.^1^^,^^2^ Unilateral spreader-extension grafts,^3^ asymmetric spreader grafts^4^ were described to restore the integrity of septal L-struts. However, relatively weak cartilage grafts (obtained from severe deviated cartilaginous septum itself) cannot maintain adequate septal support. A more rigid framework is necessary against deforming forces during treatment period. However alloplastic materials were used as spreader grafts for more rigid stabilization,^5^ there has been concerns about using alloplastic materials. Ribs are shaped as spreader grafts but rib cartilage has an unpredictable tendency to bend overtime.^6^

Other disadvantages of rib harvest comprise risk of pneumothorax. Different bone graft sources were described for nasal reconstruction.^7^^-^^9^ Fibula is a longitudinal bone as radius,^8^ and it is not curved as olecranon^9^ and calvarial bone.^10^ Lateral aspect of fibula provides a source of straight framework when shaped properly. The dorsal strut was placed as tongue in groove technique, and it was interlocked with caudal strut as “key in the keyhole” pattern. L-strut shaped bone graft supports the realigned caudal-dorsal septum position. Fibular bone graft did not obstruct airway for its delicate shape. We believe that fibular bone grafts offer a long lasting support in treatment of selected nose deformities. 

## CONFLICT OF INTEREST

The authors declare no conflict of interest.
